# Investigating the Feasibility, Acceptability and Efficacy of Using Modified-Written Exposure Therapy in the Aftermath of a Terrorist Attack on Symptoms of Posttraumatic Stress Disorder Among Afghan Adolescent Girls

**DOI:** 10.3389/fpsyt.2022.826633

**Published:** 2022-04-08

**Authors:** Sayed Jafar Ahmadi, Zeinab Musavi, Nasratullah Samim, Masooma Sadeqi, Laura Jobson

**Affiliations:** ^1^Counselling Department, Psychology Faculty, Kabul Education University, Kabul, Afghanistan; ^2^Behrawan Research and Psychological Services Organization, Kabul, Afghanistan; ^3^Turner Institute for Brain and Mental Health, School of Psychological Services, Monash University, Melbourne, VIC, Australia

**Keywords:** writing for recovery, written exposure therapy, posttraumatic stress disorder, cognitive behavior therapy, Afghan adolescents

## Abstract

**Background:**

The aim of this study was to assess the efficacy, acceptability and feasibility of using modified written exposure therapy (m-WET) to treat symptoms of posttraumatic stress disorder (PTSD) in Afghan adolescent girls in the aftermath of a terrorist attack.

**Methods:**

120 Afghan (Hazara) adolescent girls who had been exposed to the Sayed al-Shuhada school terrorist attack were randomly assigned to the m-WET (*n* = 40), trauma-focused cognitive behavior therapy (TF-CBT) (*n* = 40), or control groups (*n* = 40). m-WET involved five consecutive daily group sessions where participants simply wrote about the terrorist attack including thoughts and feelings. TF-CBT was an intensive five-session group intervention. The control group had no additional contact. The trial was undertaken at a local non-government organization in Kabul. The primary analysis was comparing PTSD symptoms (Child Revised Impact of Event Scale-13) in the three groups at post-intervention and three-month follow-up.

**Results:**

Overall, participant and facilitator satisfaction with m-WET was high. Acceptability of m-WET was relatively high, with 15% drop-out in the m-WET group and all m-WET sessions were attended. While the groups did not differ significantly in PTSD symptoms at baseline, the m-WET group had significantly lower levels of PTSD symptoms compared to the control group at post-intervention and follow-up. There was no significant difference between the m-WET and TF-CBT groups.

**Conclusion:**

The findings suggest m-WET may be promising intervention for the treatment of PTSD among adolescent girls in humanitarian settings. Further research in the area is warranted.

## Introduction

Afghanistan has endured a long-history of armed conflict, poverty, and social injustice (1). This has significantly impacted on the mental health of Afghan youth and resulted in sustained psychological trauma and substantial mental health burden (1–3). Over half the Afghan population suffer from anxiety, posttraumatic stress disorder (PTSD), or depression (1) and conflict and insecurity continue to impact negatively on Afghan adolescents’ mental and physical health (1, 4). Research has shown high levels of psychological distress and poor mental health among Afghan youth, including PTSD symptoms (5), with symptoms severely affecting long-term cognitive, emotional, social and academic/vocational functioning (6).

While Afghan youth have high levels of psychological distress, within Afghanistan the Hazara as a group have endured some of the most severe forms of oppression and persecution with significant impacts on the wellbeing of young people (7). The Hazara are one of the largest ethnic and religious minority groups in Afghanistan (8) and have a long history of oppression and persecution because of their ethnicity and religion (7). While there has been significant research into the psychological adjustment of young refugees, very little research has focused on the wellbeing of young Hazaras (7). Moreover, of the few studies conducted most have explored the well-being of young Hazaras with refugee backgrounds living in high-income countries (7, 9). Very little, if any, psychological research has been conducted with Hazara youth residing in Afghanistan.

Compounding this, on the 8th May 2021 (26 Ramadan 1442 AH) terrorist attacks (a car bombing and two improvised explosive device blasts) occurred at the entrance of Sayed al-Shuhada school in Kabul (10). Sayed al-Shuhada is located in a predominantly Shia Hazara area. At least 90 people died and 240 were injured as a result of the attack [(10); Reuters, (11)], with the majority of the casualties being girls between 11 and 15 years old (10), which is of relevance as girls in Afghanistan face additional challenges [e.g., difficulties accessing education, marital expectations, family violence^1^ and mental health concerns (1). For the survivors, the effects of these bombings compounded the trauma previously experienced by these youth. While the school reported that these girls were experiencing great psychological distress, it is difficult for youth in Afghanistan to access evidence-based psychosocial interventions (12–14).

Mental health has been identified as a humanitarian priority and addressing psychological concerns in the critical developmental period of adolescence is imperative (6, 15, 16). Nevertheless, the needs of adolescents in low-income countries and humanitarian settings has not received sufficient attention in the fields of psychology and psychiatry (17, 18). Very few adolescents in low-income countries, such as Afghanistan, receive evidence-based interventions, due to high costs, limited mental health services and a shortage of skilled professionals (12–14). Additionally, most evidence-based trauma-focused interventions are complex, high-intensity, require highly-skilled therapists, are expensive to deliver, and associated with high drop-outs (19, 20). Thus, they are difficult to implement in humanitarian contexts. In Afghanistan the mental health system is failing to meet community needs due to poor governance, limited government spending, disorganization, prolonged armed conflict, and a shortage of professional health workers (1, 21, 22). Therefore, despite the mental health concerns experienced by Afghan adolescents, most do not receive adequate mental health care (1), with only about 10% of those with mental health problems in Afghanistan receiving effective psychosocial therapy (1). This lack of access to evidence-based psychosocial interventions is highly problematic.

Mental health interventions need to be designed for relevance in humanitarian settings due to unpredictable security situations, on-going violence, and very limited access to basic services. Psychological interventions need to be low intensity and widely and freely available. Community accessible psychological services are urgently needed to assist Afghan adolescents who have been exposed to conflict, war and suicide bombings in order to prevent long-term individual and societal consequences (1, 21). There has been an urgent call for mental health services to focus on adolescents and examine the efficacy of less expensive options for providing mental health services (1).

In response to this, we conducted a pilot randomized control trial examining the efficacy, acceptability and feasibility of using a modified written exposure therapy (m-WET) in reducing symptoms of PTSD in adolescents in the aftermath of a terrorist attack. Our intervention was a modified form of written exposure training and writing for recovery (23–25). Modifications included changes to session timing, writing instructions, psychoeducation material and sessions being administered in group-settings (see Supplementary Material for further details). M-WET is low-intensity and was designed to be delivered by community facilitators with minimal training. It involved five group sessions, with 5–8 adolescents in each group. In the sessions the facilitator simply read the instructions and the participants complete a 30-min writing task; writing about the terrorist attack including thoughts, feelings and impacts on life. m-WET was compared to trauma-focused cognitive behavior therapy (TF-CBT; an intensive psychological intervention delivered by a specialist clinical psychologist) and a waitlist control group.

We first aimed to investigate the feasibility and acceptability of m-WET for adolescent girls following a terrorist attack in a humanitarian setting (Objective 1). Second, we aimed to investigate the efficacy of m-WET at post-intervention in reducing symptoms of PTSD in adolescents in the aftermath of a terrorist attack (Objective 2). Finally, we aimed to investigate whether any improvements were maintained at three-month follow-up (Objective 3). We predicted that adolescents in the m-WET group would have significantly fewer PTSD symptoms post-intervention and at three-month follow-up than the control group. The comparisons between the m-WET and TF-CBT groups were exploratory.

## Method

### Design

We used a randomized control trial. Following baseline assessment, participants were randomly allocated to one of the three trial arms using a computer-generated sequence. All assessments were conducted by researchers who were blind to the aims and hypotheses of the trial and group allocation of participants. Assessments occurred at pre-intervention, post-intervention and three-months follow-up. The data was collected at Behrawan Research and Psychology Services (a local NGO in Kabul). The intervention sessions occurred at Sayed al-Shuhada School and sessions were held at the same time each day (morning). The trial protocol can be accessed by contacting the authors.

### Participants

**FIGURE 1:**
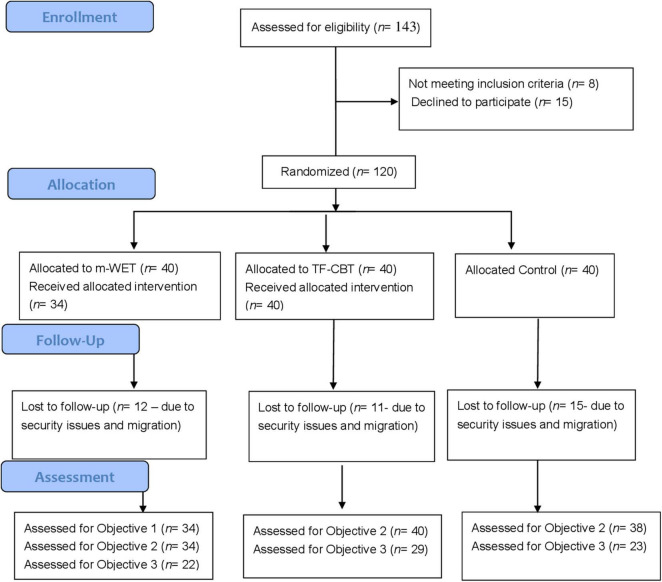
CONSORT flow diagram.

Participants included 120 Hazara adolescent girls, aged between 12 and 18 years old (School Grade 6–12), who had been exposed to the May terrorist attack at Sayed al-Shuhada school. Inclusion criteria were: (1) an adolescent who attended Sayed al-Shuhada, (2) being exposed to the bombing on 8 May, 2021, (3) experiencing heightened PTSD symptoms as indexed by scoring above 25 on the Child Revised Impact of Event Scale [CRIES; (26)], and (4) able to complete the tasks in Dari or Pashto. This sample size aligns with current approaches for estimating sample size for pilot and feasibility studies [e.g., (27)]. Figure 1 depicts the CONSORT Flow Diagram (see Supplementary Material for CONSORT checklist).

### Measures

#### Child Revised Impact of Event Scale [CRIES; (26)]

The CRIES is a 13-item self-report measure of symptoms of PTSD (28). It includes three subscales; intrusions, avoidance and arousal. The CRIES was self-completed by the adolescents. Adolescents responded to items on scales ranging from 0 (*not at* all) to 5 (*often*), with total scores ranging from 0 to 65 and higher scores indicating greater PTSD symptoms (26). The CRIES has been found to have good psychometric properties (26) and has been used with Afghan adolescents (2, 29).

### Interventions

#### Modified Written Exposure Therapy

Modified written exposure therapy was a modified form of written exposure training and writing for recovery (23–25). m-WET focused on the terrorist attack. It involved five daily group sessions, with 5–8 adolescents in each group. In the first session the purpose of m-WET was outlined. This included psychoeducation about the trauma memory, trauma-related symptoms, the impacts of war and conflict on friendships, school, family relationships, sport and hobbies, the use of maladaptive coping mechanisms, and the rationale for confronting the memory of the terrorist attack. In the five sessions, adolescents repeatedly wrote about the terrorist attack. They were encouraged to write about the details of the trauma as they remember it now (including specifics of what happened (sights, smells, sounds), what they were feeling and thinking as the attack was happening, focusing on the worst aspects of the event, how the event had touched their life, how the event might tie to other parts of their lives – such as their childhood, relationship with parents, friends, teachers, previous traumas – how the event is connected to who they would like to be in the future, and what they have learnt from the experience). Adolescents wrote about the attack for a full 30-min with no distractions or interruptions. After the 30-min the facilitator asked adolescents to finish up, thanked them for their efforts, and ensured adolescents were ready to leave. The adolescents left their workbooks behind for the next session. Between sessions the facilitators read over the narratives to ensure the adolescents had understood the task and were engaging appropriately. However, in line with WET, the facilitators did not discuss the written narratives with the adolescents (23, 24). At the end of m-WET the facilitators retained the narratives. m-WET was delivered by facilitators with minimal training- each facilitator received 8 h of training (two 4 h/session).

#### Trauma-Focused Cognitive Behavior Therapy

Trauma-focused cognitive behavior therapy (TF-CBT) was a five-session group intervention conducted by a highly-qualified clinical psychologist, with specialist training in TF-CBT. Session 1 included psychoeducation about the impact of trauma and common posttraumatic reactions. Session 2 focused on relaxation, stress-management skills and coping with emotions. Sessions 3 and 4 focused on cognitive coping and cognitive processing. It included illustrating the relationships between thoughts, feelings and behaviors, identifying and modifying inaccurate or unhelpful thoughts about the terrorist attack and *in vivo* mastery of traumatic reminders. Session 5 provided a review of the intervention.

### Feasibility and Acceptability

We assessed feasibility of recruitment by determining the number of adolescents who were approached and agreed to participate in m-WET. We assessed acceptability of intervention by measuring loss to follow-up (at both post-test and three-month follow-up). We determined acceptability of treatment based on the number of m-WET sessions attended by adolescents in the m-WET arm of the trial. At the end of Session 5, adolescents and facilitators provided feedback on the intervention and were asked: (1) what they liked about m-WET, (2) what they did not like about m-WET, and (3) how m-WET could be improved. Therapist adherence to sessions was determined by clinical supervision with a clinical psychologist where each session was reviewed regarding adherence to the manual.

### Procedure

Ethical approval was obtained from the Afghan Ministry of Health. The trial was conducted at least 1 month following the terrorist attack. Information sheets and consent forms were given to the adolescent and their parents/guardians. Participants were tested individually by researchers who were blind to group status on three occasions: pretraining, post-training and three-month follow-up. The pretraining assessment consisted of the CRIES. Following this, we randomly allocated participants to the intervention groups or the control group (which had no additional contact). The CRIES and interventions were presented in Pashto or Dari [the CRIES was translated and back-translated using gold-standard procedures, (30)]. At post-training and at three-month follow-up, the CRIES was re-administered.

### Data Analysis Plan

We reported information about feasibility of recruitment, acceptability of the intervention, and satisfaction with m-WET (Objective 1). All statistical analyses were conducted using IBM SPSS Statistics 27. Prior to hypothesis testing, data cleaning was conducted. There was no missing data. Several of the variables, particularly for the CBT group, did not meet the assumptions for normality (assessed using Shapiro-Wilk test). As attempts to transform the data did not improve normality, bootstrapping was applied (31). All variables, with the exception of PSTD follow-up data, met the assumption of homogeneity of variance (assessed using Levene’s test) (31). We also conducted the PTSD follow-up analyses using non-parametric tests. A similar pattern of results emerged to that presented below (see Supplementary Material).

We first compared the three groups on baseline assessment data. To test the hypotheses, we used Analysis of Covariances (ANCOVAs) with PTSD symptoms as the dependent variable; we investigated group differences at post-training (Objective 2) and three-month follow-up (Objective 3) with baseline PTSD symptoms included as a covariate. We also conducted two exploratory Multivariate Analysis of Covariance Analyses (MANCOVAs) where the PTSD symptom clusters were the outcome variables for both post-training and follow-up and baseline data was included as covariates.

## Results

### Group Characteristics

Group characteristics are presented in Table 1. The three groups did not differ significantly in age, *F*(2,110) = 1.02, *p* = 0.37, η_p_^2^ = 0.02, number of people in family, *F*(2,85) = 0.57, *p* = 0.77, η_p_^2^ = 0.01, birth order, χ^2^ (4,*N* = 105) = 1.83, *p* = 0.77, self-reported economic status, χ^2^ (2,*N* = 109) = 0.57, *p* = 0.75, whether the adolescent’s father was alive, χ^2^(2, *N* = 110) = 0.72, *p* = 0.70, or father’s, χ^2^(6, *N* = 89) = 4.99, *p* = 0.55, and mother’s level of education, χ^2^(2, *N* = 92) = 4.23, *p* = 0.12. The majority of the sample reported that their parents were illiterate and all participants reported that their mother was alive. The groups also did not differ significantly in their previous exposure to a terrorist attack, χ^2^(2, *N* = 111) = 2.67, *p* = 0.26, or currently being on medication for psychological concerns, χ^2^(2, *N* = 101) = 5.24, *p* = 0.07.

**TABLE 1:** Group characteristics.

	m-WET	TF-CBT	Control
***Demographics*** Age (years)	15.74 (2.16)	15.85 (2.13)	16.37 (1.78)
Number of people in family	8.03 (1.99)	7.63 (2.11)	8.18 (1.95)
Place in family (eldest child:middle child: youngest child)	6:12:15	8:17:11	6:16:14
Father alive (alive:deceased)	30:2	36:4	36:2
Mother alive (alive:deceased)	32:0	40:0	38:0
Father’s education (illiterate: high school graduate: diploma graduate: Bachelor degree or Masters degree)	29:2:0:0	27:0:1:1	27:1:0:1
Mother’s education (illiterate: high school graduate)	31:0	28:2	31:0
Self-rated economic status (low:middle)	24:11	24:13	27:10
Previous terrorists attack exposure (yes:no)	5:29	11:28	6:32
Currently on medication (yes:no)	8:21	12:36	18:16
***PTSD symptoms*** **Baseline**			
Intrusion	11.00 (5.50)	10.83 (5.72)	13.21 (3.50)
Avoidance	11.99 (6.40)	10.43 (6.46)	12.71 (4.46)
Arousal	14.01 (6.60)	15.16 (7.58)	18.81 (4.63)
**Post-Intervention**			
Intrusion	8.83 (4.23)	10.15 (4.70)	11.71 (4.84)
Avoidance	11.60 (4.72)	10.66 (6.03)	13.24 (5.14)
Arousal	11.69 (5.09)	14.68 (4.74)	16.50 (4.49)
**Follow-up**			
Intrusion	9.64 (5.09)	9.55 (4.82)	13.52 (3.82)
Avoidance	11.27 (6.03)	11.79 (6.13)	13.17 (4.65)
Arousal	13.14 (6.10)	15.93 (6.06)	19.43 (4.83)

*PTSD, posttraumatic stress disorder. m-WET, modified written exposure therapy. TF-CBT, trauma-focused cognitive behavior therapy. The three groups did not differ significantly at baseline in terms of PTSD symptoms, with the exception of baseline arousal, F(2,109) = 5.66, p < 0.01. All exploratory analyses controlled for baseline symptoms.*

### Feasibility and Acceptability

A total of 143 adolescents were screened for eligibility through Sayed al-Shuhada school; 15 participants declined to participate and eight participants were excluded due to not meeting our inclusion criteria (see Figure 1). Acceptability of randomization was high; no participants dropped out after they learned their randomization status. Acceptability of m-WET and TF-CBT were relatively high; six adolescents (15%) in m-WET dropped out, no adolescents in TF-CBT dropped out, and two adolescents in the control group dropped out. Of the remaining participants, all attended all sessions of m-WET or TF-CBT. However, 38 participants (m-WET *n* = 22, TF-CBT *n* = 29, Control *n* = 23) were lost to follow-up. This was due to security concerns and migration reasons, particularly as follow-up period aligned with the Taliban gaining control of Afghanistan. Those lost to follow-up did not differ significantly from those who completed the study in terms of demographics and baseline PTSD symptoms, with the exception of age; those lost to follow-up were significantly older (see Supplementary Table 2).

The adolescents and facilitators who provided qualitative feedback on m-WET reported that initially they did not believe that writing would be so effective. The adolescents noted that at first, they were afraid to face their own experiences and avoided it, but now they have the courage to face it. They also noted that they are now more focused and pay more attention to the details of events. The facilitators shared this view and noted that the m-WET participants were calmer than the adolescents in the TF-CBT after the interventions. The facilitators also noted that within the final sessions the incidence of negative emotions was lower in the m-WET group than the TF-CBT. The facilitators stated that the positive aspect of m-WET was that it connects the past to the future and allows adolescents to learn from the past in the present and plan for the future. Additionally, m-WET acts as a writing skill training and thus, after m-WET participants felt that they had learnt a new skill while also recovering. The facilitators noted that some of the adolescents continued to write and even had their stories ready for publication and sometimes sent their future stories in the form of a letter to the facilitators after the group had finished. Adolescents also noted that following m-WET they had encouraged their friends and family to write about the attack. Overall, satisfaction with m-WET was high. Therapists adherence was high; all therapists adhered to the m-WET manual. Therapists reported that they could readily get through the material in 30–45-min sessions. There were no important harms or unintended effects in either group.

### Baseline

At baseline, the three groups did not differ significantly in PTSD symptoms, *F*(2,109) = 0.02, *p* = 0.98, η_p_^2^ < 0.001.

### Post-intervention

**FIGURE 2:**
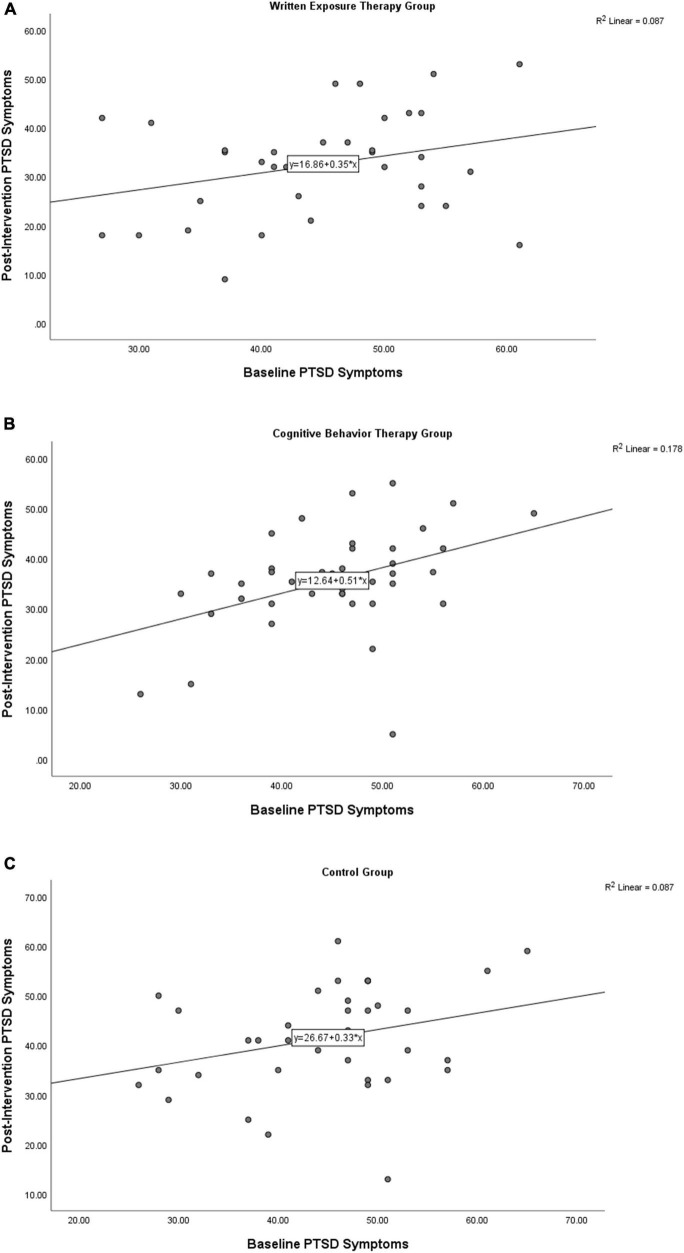
Scatterplots for the Baseline and Post-Intervention Posttraumatic Stress Disorder (PTSD) symptoms for the written exposure group **(A)**, cognitive behavior therapy group **(B)** and control group **(C)**.

**FIGURE 3:**
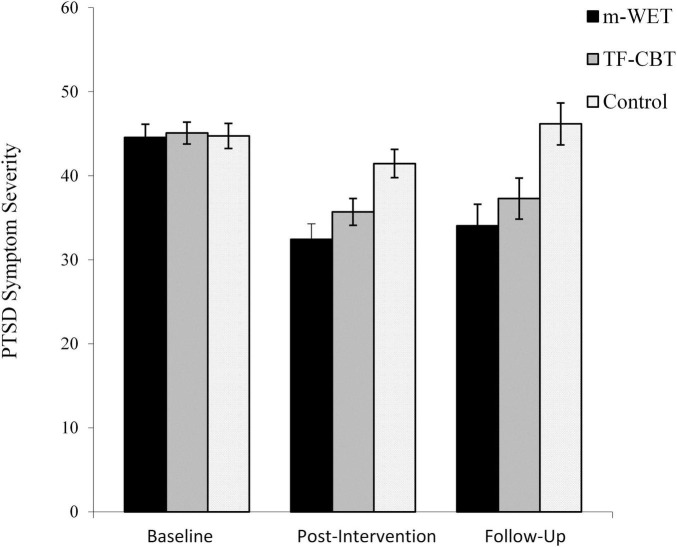
Depicting posttraumatic stress (PTSD) symptom severity at baseline, post-intervention and 3-month follow-up for the modified- Written Exposure Therapy (m-WET), trauma-focused-cognitive behavior therapy (TF-CBT), and control groups.

Baseline and post-intervention PTSD symptoms were significantly associated for the CBT group, *r*_*s*_(39) = 0.40, *p* = 0.01, 95%CI[0.12–0.66], but not the control, *r*_*s*_(37) = 0.24, *p* = 0.14, 95%CI[−0.15–0.57] or m-WET groups, *r*_*s*_(33) = 0.25, *p* = 0.16, 95%CI[−0.15–0.63] (Figure 2). At post-intervention the three groups differed significantly, *F*(2,108) = 7.87, *p* = 0.001, η_p_^2^ = 0.13 (Figure 3). Follow-up analyses revealed the m-WET group had significantly lower PTSD symptom severity than the control group, *F*(1,69) = 14.04, *p* < 0.001, η_p_^2^ = 0.17. The TF-CBT group also had significantly lower PTSD symptom severity than the control group, *F*(1,75) = 7.33, *p* < 0.01, η_p_^2^ = 0.09. The TF-CBT and m-WET groups did not differ significantly, *F*(1,71) = 1.86, *p* = 0.18, η_p_^2^ = 0.03.

### Three-Month Follow-Up

At follow-up the three groups differed significantly, *F*(2,70) = 7.11, *p* = 0.002, η_p_^2^ = 0.13. The m-WET group had significantly lower PTSD symptom severity than the control group, *F*(1,42) = 20.78, *p* < 0.001, η_p_^2^ = 0.33. The TF-CBT group also had significantly lower PTSD symptom severity than the control group, *F*(1,49) = 7.46, *p* = 0.009, η_p_^2^ = 0.13. The TF-CBT and m-WET groups did not differ significantly, *F*(1,48) = 0.94, *p* = 0.34, η_p_^2^ = 0.02.

### Exploratory Analysis

Correlation data, figures depicting group means and scatterplots for individual symptom clusters are presented in Supplementary Material (Supplementary Table 1 and Supplementary Figures 1, 2, respectively). When examining symptoms at post-intervention, there was a significant effect of group, Wilks’ Lambda = 0.86, *F*(6,208) = 2.65, *p* = 0.02, η_p_^2^ = 0.07. The three groups differed significantly on intrusion, *F*(2,106) = 3.50, *p* = 0.03, η_p_^2^ = 0.06, and arousal symptoms, *F*(2,106) = 5.65, *p* < 0.01, η_p_^2^ = 0.10, but not avoidance symptoms, *F*(2,106) = 2.59, *p* = 0.08, η_p_^2^ = 0.05. Follow-up analyses showed the m-WET group had significantly lower intrusion symptom severity than the control group, *F*(1,69) = 5.71, *p* = 0.02, η_p_^2^ = 0.08, but did not differ significantly from the TF-CBT group, *F*(1,71) = 1.82, *p* = 0.18, η_p_^2^ = 0.03. The TF-CBT and control groups did not differ significantly in intrusion symptoms, *F*(1,75) = 0.25, *p* = 0.62, η_p_^2^ = 0.003. The m-WET group had significantly lower arousal symptom severity than the control, *F*(1,69) = 12.16, *p* = 0.001, η_p_^2^ = 0.15, and TF-CBT groups, *F*(1,71) = 6.20, *p* = 0.02, η_p_^2^ = 0.08. The TF-CBT and control groups did not differ significantly, *F*(1,75) = 0.18, *p* = 0.68, η_p_^2^ = 0.002.

When examining symptoms at follow-up, there was a significant effect of group, Wilks’ Lambda = 0.82, *F*(6,132) = 2.33, *p* = 0.04, η_p_^2^ = 0.10. The three groups differed significantly on intrusion, *F*(2,68) = 4.24, *p* = 0.02, η_p_^2^ = 0.11, and arousal symptoms, *F*(2,68) = 4.40, *p* = 0.02, η_p_^2^ = 0.12, but did not differ significantly on avoidance symptoms, *F*(2,68) = 1.15, *p* = 0.32, η_p_^2^ = 0.03. Follow-up analyses showed that the m-WET group had significantly lower intrusion symptom severity than the control group, *F*(1,42) = 6.83, *p* = 0.01, η_p_^2^ = 0.14, but did not differ significantly from the TF-CBT group, *F*(1,48) < 0.001, *p* > 0.99, η_p_^2^ < 0.001. The TF-CBT group had significantly lower intrusion symptom severity than the control group, *F*(1,49) = 7.13, *p* = 0.01, η_p_^2^ = 0.13. The m-WET group had significantly lower arousal symptom severity than the control group, *F*(1,42) = 8.76, *p* = 0.005, η_p_^2^ = 0.17, but did not differ significantly from the TF-CBT group, *F*(1,48) = 2.42, *p* = 0.13, η_p_^2^ = 0.05. The TF-CBT and control groups did not differ significantly in terms of arousal symptoms, *F*(1,49) = 2.12, *p* = 0.15, η_p_^2^ = 0.04.

## Discussion

This study examined the feasibility, acceptability and efficacy of m-WET in treating PTSD symptoms in Afghan adolescent girls in the aftermath of a terrorist attack. First, we found m-WET was a feasible and acceptable psychological intervention for adolescent girls in the aftermath of a terrorist attack. Adolescents and facilitators indicated m-WET would be a useful intervention for other trauma-exposed youth. Second, at post-intervention and three-month follow-up the m-WET group had significantly lower PTSD symptom severity than the control group. The TF-CBT group also had significantly lower PTSD symptom severity than the control group and the TF-CBT and m-WET groups did not differ significantly. Third, when considering PTSD symptom clusters at post-training and follow-up the three groups differed significantly on intrusion and arousal symptoms, but not on avoidance symptoms. Specifically, at both post-training and follow-up the m-WET group had significantly lower intrusion and arousal symptom severity than the control group. The m-WET and TF-CBT groups did not differ significantly, with the exception of the m-WET group having significantly lower arousal symptoms at post-training than the TF-CBT group.

Our findings are promising. m-WET appears to be a feasible and acceptable intervention that can reduce PTSD symptoms in adolescents living in a humanitarian setting. m-WET is a low intensity, community accessible intervention that can be delivered by those with minimal training, is acceptable to adolescents and facilitators, and has the potential to be made widely and freely available for the community. This is important in the context of Afghanistan; a country with despite a long-history of armed conflict, poverty, social injustice and immense mental health concerns [e.g., (1–3)] has exceptionally limited mental health services (1, 12–14). Our findings also indicate that m-WET may be an intervention that has utility for adolescents in the aftermath of a terrorist attack in low-income countries; an area that has not received sufficient attention in the fields of psychology and psychiatry (17, 18). Additionally, the study demonstrated that the delivery of brief, group TF-CBT by a clinical psychologist also reduced PTSD symptoms.

It is worth noting m-WET may benefit from greater rationale regarding the writing process in Session1 and in promotion of the intervention, as the adolescents and facilitators were initially skeptical about the writing tasks. Further, as adolescents noted that they were initially afraid to write about their experiences, it would be worth further normalizing these feelings in Session 1. Finally, it was observed that some of the younger girls and those with lower levels of education wrote less because of their writing skill level and m-WET is limited to those who are literate. Future research should investigate the cost-effectiveness of these interventions, conduct larger randomized control trials and examine the feasibility and efficacy of m-WET in other humanitarian settings.

The limitations of the study are as follows. First, the study would have been improved had a wider range of symptoms (depression, anxiety, quality of life) been assessed. However, given the current security situation in Kabul, assessment sessions had to be as brief as possible. However, future research using different samples should include further psychosocial measures. Second, it is worth noting that while m-WET and TF-CBT reduced PTSD symptoms, symptoms were still elevated at post-training and follow-up indicating further sessions or modules may be needed to further reduce symptoms. Additionally, the groups did not differ from the control group on avoidance symptoms. Hence, specific further work addressing avoidance symptoms may benefit the intervention. Third, we deliberately focused on adolescents with high levels of PTSD symptoms rather than a particular psychiatric diagnosis. This is consistent with similar studies focusing on the development of low intensity interventions (6). Fourth, a considerable number of participants were lost to follow-up. This was influenced by security concerns and migration reasons associated with our follow-up period aligning with the Taliban gaining control of Afghanistan. Therefore, our follow-up findings (Objective 3) should be interpreted with caution. Finally, our study focused on Hazara adolescent girls living Kabul. Thus, the generalizability of our findings needs to be considered and further research is needed. In sum, our findings support m-WET as an active training intervention that can successfully improve PTSD symptoms to a level equivalent to TF-CBT and such improvements appear to be maintained at follow-up.

## Data Availability Statement

The raw data supporting the conclusions of this article will be made available by the authors, without undue reservation.

## Ethics Statement

The studies involving human participants were reviewed and approved by Afghan Ministry of Health. Written informed consent to participate in this study was provided by the participants’ legal guardian/next of kin.

## Author Contributions

SJA, ZM, NS, MS, and LJ: conceptualization, methodology, writing—review and editing, and project administration. SJA and LJ: formal analysis. SJA, ZM, NS, and MS: investigation. LJ, SJA, and ZM: resources. SJA, ZM, and LJ: writing—original draft preparation. SJA: supervision. All authors have read and agreed to the published version of the manuscript.

## Conflict of Interest

The authors declare that the research was conducted in the absence of any commercial or financial relationships that could be construed as a potential conflict of interest.

## Publisher’s Note

All claims expressed in this article are solely those of the authors and do not necessarily represent those of their affiliated organizations, or those of the publisher, the editors and the reviewers. Any product that may be evaluated in this article, or claim that may be made by its manufacturer, is not guaranteed or endorsed by the publisher.

## Footnotes

1 Ahmadi SJ, Ayubi B, Fateh MA, Musavi Z. *Suicidal Thoughts Among Afghan Women: Rating and Relationship with Family Conflicts and Violence*. (2021).
