# Supplementary material for: Investigating the Feasibility, Acceptability and Efficacy of Using Modified-Written Exposure Therapy in the Aftermath of a Terrorist Attack on Symptoms of Posttraumatic Stress Disorder Among Afghan Adolescent Girls
Source: Front Psychiatry. 2022 Apr 8;13:826633. doi: 10.3389/fpsyt.2022.826633 (PMC9027104; doi:10.3389/fpsyt.2022.826633)

SUPPLEMENTARY MATERIAL

Supplemental Information

Outline of Intervention Modifications

Writing for Recovery is designed to be conducted over three separate days for two short (15 minute) periods of writing each day (with brief rest periods between sessions). The days can be consecutive or spaced further apart. Written exposure therapy (WET) recommends five weekly sessions including 30 minutes of writing. In modified Written Exposure Therapy (m-WET) each session is 30 minutes and it is more intensive (five sessions over five days). This intensive approach was decided upon due to security reasons in Kabul, the adolescents' motivation to engage in an intensive intervention, and the girls being able to access the intervention at the school during this week. m-WET included a modification of the WET psychoeducation material so it would be more developmentally appropriate and relevant for adolescents. Additionally, the session content was slightly more instructive as facilitators encouraged the adolescents to write about the details of the trauma as they remember it now and also to write about what they were feeling and thinking as the attack was happening, focusing on the worst aspects of the event, how the event had touched their life, how the event might tie to other parts of their lives – such as their childhood, relationship with parents, friends, teachers, previous traumas – how the event is connected to who they would like to be in the future, and what they have learnt from the experience. Additionally, while WET is typically an individual intervention, m-WET was administered in group-settings.

Non-Parametric Analyses for Three-Month Follow-Up PTSD Symptom Data

At follow-up the three groups differed significantly in terms of PTSD symptom severity, Kruskal-Wallis H (df=2)= 12.17, p= .002. Follow-up analyses revealed the m-WET group (Mean Rank = 16.20) had significantly lower PTSD symptom severity than the control group (Mean Rank = 29.50), Mann-Whitney U= 103.50, Z= 3.40, p= .001. The TF-CBT (Mean Rank = 22.00) group also had significantly lower PTSD symptom severity than the control group (Mean Rank = 32.17), Mann-Whitney U= 203.00, Z= 2.41, p= .02. The TF-CBT (Mean Rank = 28.17) and m-WET (Mean Rank = 23.14) groups did not differ significantly, Mann-Whitney U= 2.56, Z= 1.20, p= .23.

Supplemental Table 1

CONSORT 2010 checklist of information to include when reporting a pilot or feasibility trial*

Section/Topic	Item No	Checklist item	Reported on page No
**Title and abstract**			
	1a	Identification as a pilot or feasibility randomised trial in the title	1
	1b	Structured summary of pilot trial design, methods, results, and conclusions (for specific guidance see CONSORT abstract extension for pilot trials)	1
**Introduction**			
Background and objectives	2a	Scientific background and explanation of rationale for future definitive trial, and reasons for randomised pilot trial	2-3
	2b	Specific objectives or research questions for pilot trial	3
**Methods**			
Trial design	3a	Description of pilot trial design (such as parallel, factorial) including allocation ratio	3
	3b	Important changes to methods after pilot trial commencement (such as eligibility criteria), with reasons	N/A
Participants	4a	Eligibility criteria for participants	4
	4b	Settings and locations where the data were collected	4
	4c	How participants were identified and consented	4 and 5
Interventions	5	The interventions for each group with sufficient details to allow replication, including how and when they were actually administered	4-5
Outcomes	6a	Completely defined prespecified assessments or measurements to address each pilot trial objective specified in 2b, including how and when they were assessed	4
	6b	Any changes to pilot trial assessments or measurements after the pilot trial commenced, with reasons	N/A
	6c	If applicable, prespecified criteria used to judge whether, or how, to proceed with future definitive trial	N/A
Sample size	7a	Rationale for numbers in the pilot trial	4
	7b	When applicable, explanation of any interim analyses and stopping guidelines	N/A

Randomisation:			
Sequence generation	8a	Method used to generate the random allocation sequence	4
	8b	Type of randomisation(s); details of any restriction (such as blocking and block size)	4
Allocation concealment mechanism	9	Mechanism used to implement the random allocation sequence (such as sequentially numbered containers), describing any steps taken to conceal the sequence until interventions were assigned	4
Implementation	10	Who generated the random allocation sequence, who enrolled participants, and who assigned participants to interventions	4
Blinding	11a	If done, who was blinded after assignment to interventions (for example, participants, care providers, those assessing outcomes) and how	4
	11b	If relevant, description of the similarity of interventions	N/A
Statistical methods	12	Methods used to address each pilot trial objective whether qualitative or quantitative	5-6
**Results**			
Participant flow (a diagram is strongly recommended)	13a	For each group, the numbers of participants who were approached and/or assessed for eligibility, randomly assigned, received intended treatment, and were assessed for each objective	Figure 1
	13b	For each group, losses and exclusions after randomisation, together with reasons	Figure 1
Recruitment	14a	Dates defining the periods of recruitment and follow-up	3-4
	14b	Why the pilot trial ended or was stopped	N/A
Baseline data	15	A table showing baseline demographic and clinical characteristics for each group	Table 1
Numbers analysed	16	For each objective, number of participants (denominator) included in each analysis. If relevant, these numbers should be by randomised group	6-7
Outcomes and estimation	17	For each objective, results including expressions of uncertainty (such as 95% confidence interval) for any estimates. If relevant, these results should be by randomised group	6-7
Ancillary analyses	18	Results of any other analyses performed that could be used to inform the future definitive trial	N/A
Harms	19	All important harms or unintended effects in each group (for specific guidance see CONSORT for harms)	6

	19a	If relevant, other important unintended consequences	N/A
**Discussion**			
Limitations	20	Pilot trial limitations, addressing sources of potential bias and remaining uncertainty about feasibility	8
Generalisability	21	Generalisability (applicability) of pilot trial methods and findings to future definitive trial and other studies	7-8
Interpretation	22	Interpretation consistent with pilot trial objectives and findings, balancing potential benefits and harms, and considering other relevant evidence	7-8
	22a	Implications for progression from pilot to future definitive trial, including any proposed amendments	7-8
**Other information**			
Registration	23	Registration number for pilot trial and name of trial registry	N/A
Protocol	24	Where the pilot trial protocol can be accessed, if available	4
Funding	25	Sources of funding and other support (such as supply of drugs), role of funders	12
	26	Ethical approval or approval by research review committee, confirmed with reference number	5

Citation: Eldridge SM, Chan CL, Campbell MJ, Bond CM, Hopewell S, Thabane L, et al. CONSORT 2010 statement: extension to randomised pilot and feasibility trials. BMJ. 2016;355.
*We strongly recommend reading this statement in conjunction with the CONSORT 2010, extension to randomised pilot and feasibility trials, Explanation and Elaboration for important clarifications on all the items. If relevant, we also recommend reading CONSORT extensions for cluster randomised trials, non-inferiority and equivalence trials, non-pharmacological treatments, herbal interventions, and pragmatic trials. Additional extensions are forthcoming: for those and for up to date references relevant to this checklist, see www.consort-statement.org.

Supplemental Table 2

Comparison of participants who were lost to follow-up and those retained in the study at follow-up

Variable	Follow-Analysis Group (*n*=74)	Drop-out at Follow-Up Group(*n*= 38)	Statistic
Age	15.73 (2.12)	16.58 (1.70)	$t(110)= 2.14, p=.03$
Number of people in family	7.90 (2.17)	8.07 (1.69)	$t(85)= .37, p=.71$
Place in family (eldest child:middle child: youngest child)	9:33:26	11:14:11	χ^2 (*N*=104, *df*=2)= 4.55, *p* =.10
Father alive (alive:deceased)	65:6	36:2	χ^2 (*N*=109, *df*=1)= .37, *p* =.54
Mother alive (alive:deceased)	74:0	38:0	N/A
Father's education (illiterate: high school graduate: diploma graduate: Bachelor degree; Masters degree)	53:2:1:1	29:1:0:1	χ^2 (*N*=88, *df*=3)= .74, *p* =.86
Mothers's education (illiterate: high school graduate)	58:2	31:0	χ^2 (*N*=91, *df*=1)= 1.06, *p* =.30
Self-rated economic status (low:middle)	51:21	23:13	χ^2 (*N*=108, *df*=1)= .54, *p* =.46
Previous terrorists attack exposure (yes:no)	14:58	7:31	χ^2 (*N*=110, *df*=1)= .02, *p* =.90
Currently on medication (yes:no)	25:42	13:20	χ^2 (*N*=100, *df*=1)= .04, *p* =.84
Baseline Intrusion	12.01 (5.16)	11.05 (4.92)	$t(110)= .95, p=.35$
Baseline Avoidance	12.01 (5.81)	10.99 (6.00)	$t(110)= .87, p=.39$
Baseline Hyperarousal	16.76 (6.36)	14.67 (7.11)	$t(110)= 1.59, p=.12$
Baseline PTSD	44.93 (8.78)	44.74 (9.04)	$t(110)= .11, p=.91$

Note: There is some missing demographics data. PTSD= posttraumatic stress disorder

Supplemental Table 3

Spearman Rho Correlation Analyses for Posttraumatic Stress Disorder (PTSD) Symptoms at each Time Point for Each Group

	Baseline Intrusion	Baseline Avoidance	Baseline Hyperarousal	Post-Intervention Intrusion	Post-Intervention Avoidance	Post-Intervention Hyperarousal	Follow-up Intrusion	Follow-up Avoidance	Follow-up Hyperarousal
**Written Exposure Therapy Group**									
Baseline Intrusion	1.00								
Baseline Avoidance	.53** [.21-.74]	1.00							
Baseline Hyperarousal	.65** [.34-.83]	.53** [.19-.81]	1.00						
Post-Intervention Intrusion	-.12 [-.49-.28]	.09 [-.29-.43]	-.14 [-.44-.20]	1.00					
Post-Intervention Avoidance	<.001 [-.35-.33]	.41* [.08-.68]	.18 [-.17-.47]	.35* [.01-.63]	1.00				
Post-Intervention Hyperarousal	-.12 [-.45-.23]	-.05 [-.40-.32]	-.05 [-.39-.29]	.54** [.22-.78]	.32* [-.05-.67]	1.00			
Follow-Up Intrusion	-.25 [-.67-.24]	-.05 [-.49-.41]	-.25 [-.64-.20]	.22 [-.25-.66]	-.04 [-.43-.37]	.20 [-.20-.50]	1.00		
Follow-up Avoidance	.27 [-.18-.64]	.51* [.11-.78]	.47* [.06-.74]	.03 [-.37-.43]	.61** [.24-.85]	.04 [-.42-.53]	-.13 [-.54-.29]	1.00	
Follow-up Hyperarousal	-.08 [-.57-.42]	.03 [-.46-.44]	.01 [-.43-.44]	-.03 [-.46-.43]	.10 [-.38-.55]	.37 [-.06-.68]	.55** [.08-.87}	-.03 [-.57-.46]	1.00
**Cognitive Behavior Group**									
Baseline Intrusion	1.00								
Baseline Avoidance	.48** [.10-.71]	1.00							
Baseline Hyperarousal	.65** [.37-.82]	.47** [.15-.69]	1.00						

Post-Intervention	.54**	.36*	.16	1.00					
Intrusion	[.22-.79]	[.04-.63]	[-.19-.48]						
Post-Intervention	-.06	.25	-.10	.10	1.00				
Avoidance	[-.42-.27]	[-.10-.55]	[-.44-.26]	[-.25-.44]					
Post-Intervention	.49**	.24	.51**	.35*	-.09	1.00			
Hyperarousal	[.18-.75]	[-.10-.55]	[.18-.76]	[-.004-.67]	[-.36-.33]				
Follow-Up	.46*	.38*	.50**	.32	.21	.65**	1.00		
Intrusion	[.10-.72]	[.02-.65]	[.16-.76]	[-.09-.65]	[-.20-.58]	[.30-.86]			
Follow-up	-.10	.22	-.14	.05	.71**	.02	.28	1.00	
Avoidance	[-.47-.32]	[-.19-.57]	[-.49-.25]	[-.36-.45]	[.38-.91]	[-.36-.38]	[-.09-.58]		
Follow-up	.30	.21	.40*	.03	.03	.55**	.60**	.19	1.00
Hyperarousal	[-.03-.58]	[-.16-.53]	[.02-.67]	[-.37-.41]	[-.33-.37]	[.20-.79]	[.27-.83]	[-.17-.50]	
**Control Group**									
Baseline Intrusion	1.00								
Baseline	.22	1.00							
Avoidance	[-.15-.60]								
Baseline	.25	.37*	1.00						
Hyperarousal	[-.07-.54]	[.05-.63]							
Post-Intervention	.26	.15	.07	1.00					
Intrusion	[-.08-.59]	[-.19-.46]	[-.31-.44]						
Post-Intervention	-.09	.26	.03	.04	1.00				
Avoidance	[-.39-.24]	[-.07-.54]	[-.35-.38]	[-.31-.37]					
Post-Intervention	.08	.31	.41*	.66**	.09	1.00			
Hyperarousal	[-.27-.42]	[-.03-.56]	[.10-.65]	[.41-.82]	[-.26-.43]				
Follow-Up	.98**	.11	.27	.30	-.10	.08	1.00		
Intrusion	[.95-.99]	[-.41-.58]	[-.17-.63]	[-.15-.68]	[-.51-.36]	[-.38-.51]			
Follow-up	.11	.97**	.42	.22	.40	.22	.05	1.00	
Avoidance	[-.41-.55]	[.86-.99]	[-.01-.75]	[-.20-.56]	[.02-.67]	[-.21-.60]	[-.46-.50]		
Follow-up	.20	.41	.97**	.09	.03	.31	.30	.40	1.00
Hyperarousal	[-.21-.58]	[-.01-.71]	[.92-.99]	[-.40-.56]	[-.42-.46]	[-.15-.68]	[-.12-.64]	[-.04-.71]	

Note: *$p < .05$, ** $p < .001$.

Supplemental Figure 1

Written Exposure Therapy (WET), Cognitive Behavior Therapy (CBT) and Control Groups at Baseline, Post-Intervention, and Three-Month Follow-Up for Intrusion (Supplemental Figure 1a), Avoidance (Supplemental Figure 1b) and Hyperarousal Symptoms (Supplemental Figure 2c).

Supplemental Figure 1a

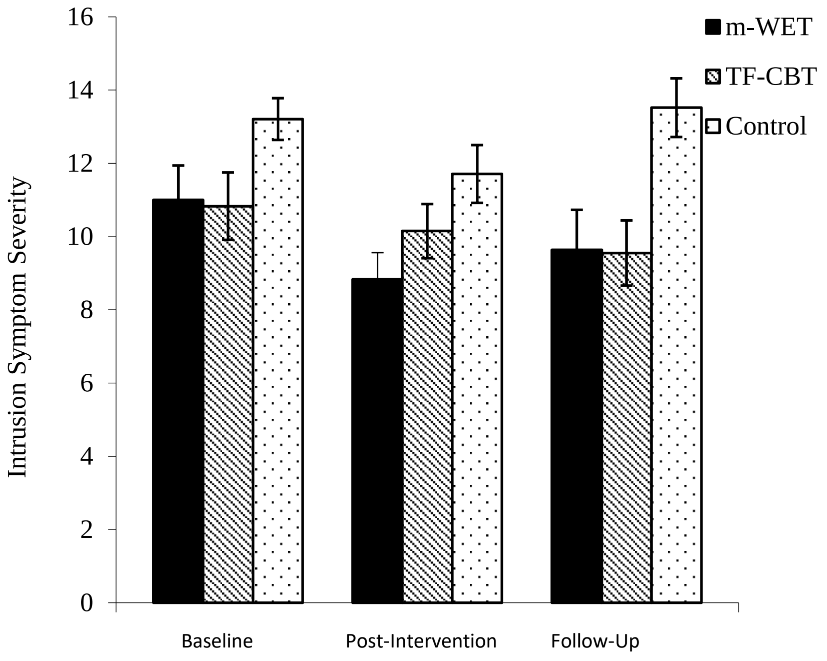

Supplemental Figure 1b

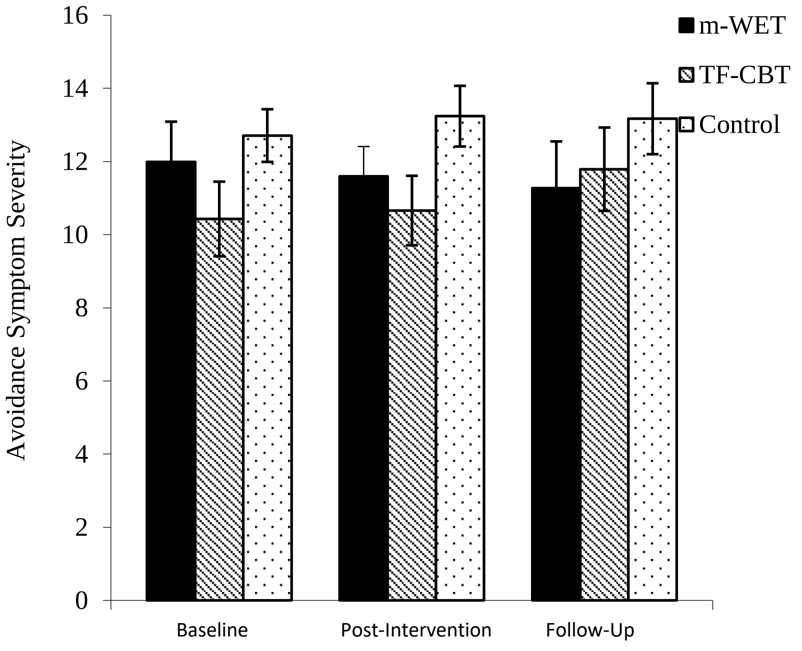

Supplemental Figure 1c

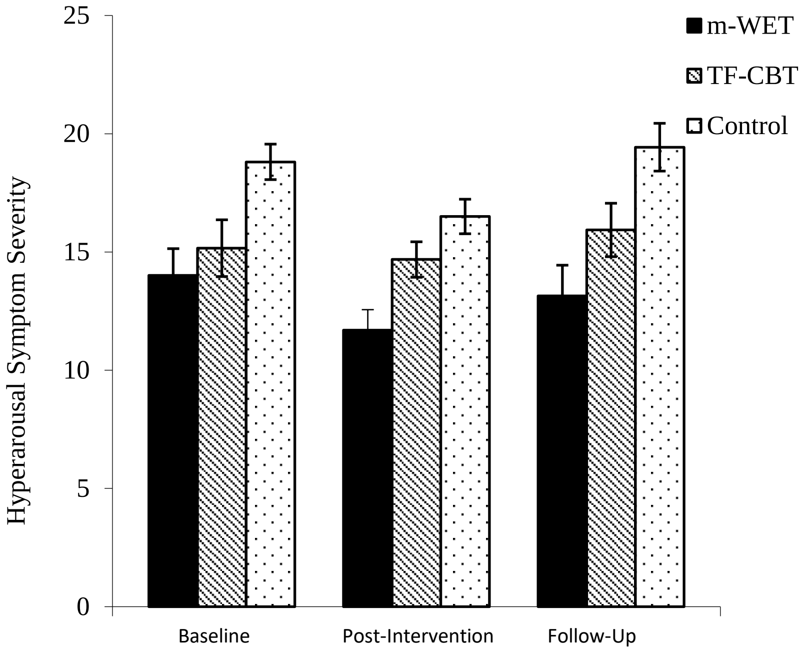

Supplemental Figure 2

Scatterplots for Written Exposure Therapy (WET), Cognitive Behavior Therapy (CBT) and Control Groups at Baseline and Post-Intervention for Intrusion Symptoms, Avoidance Symptoms and Hyperarousal Symptoms.

Intrusion Symptoms

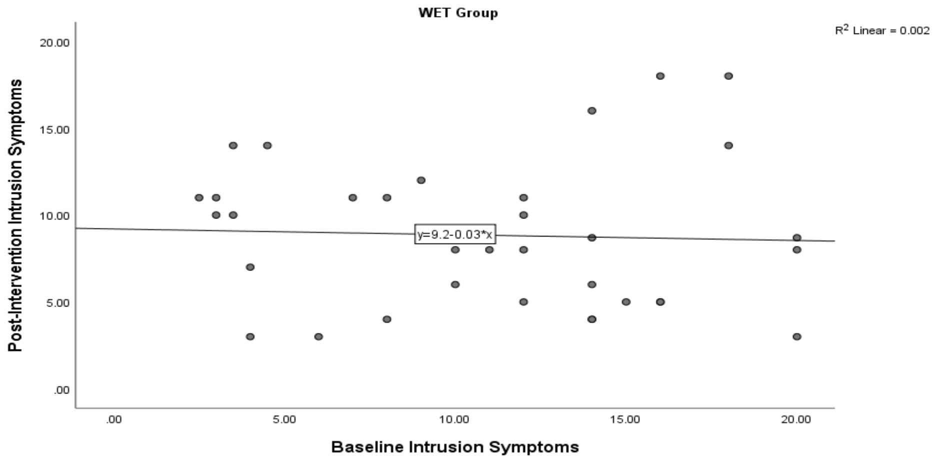

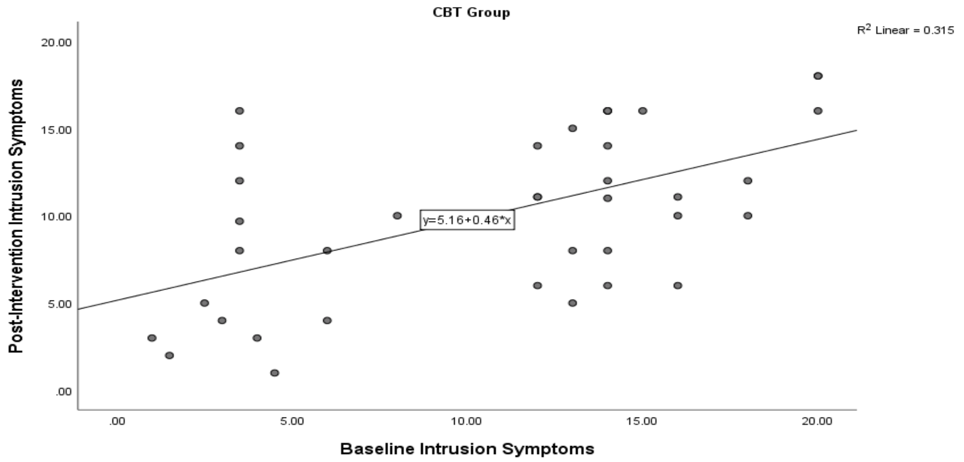

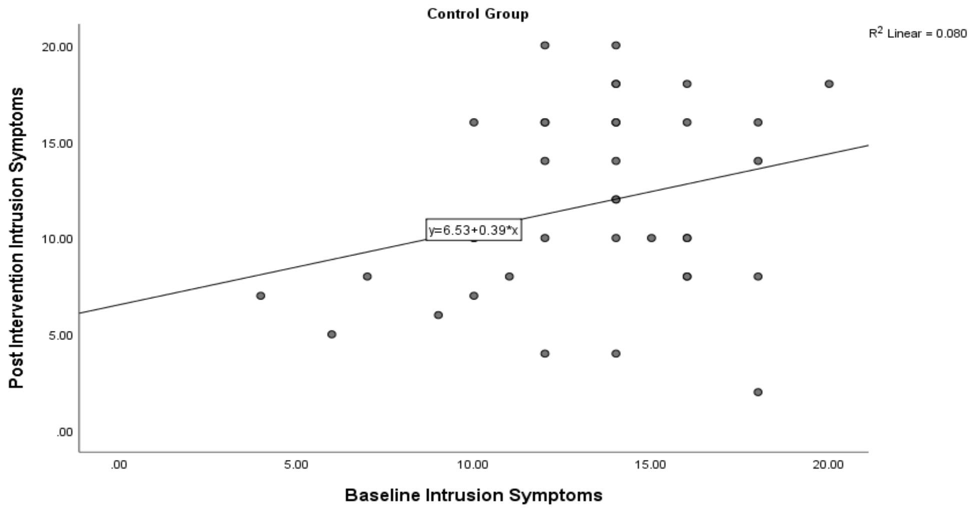

Avoidance Symptoms

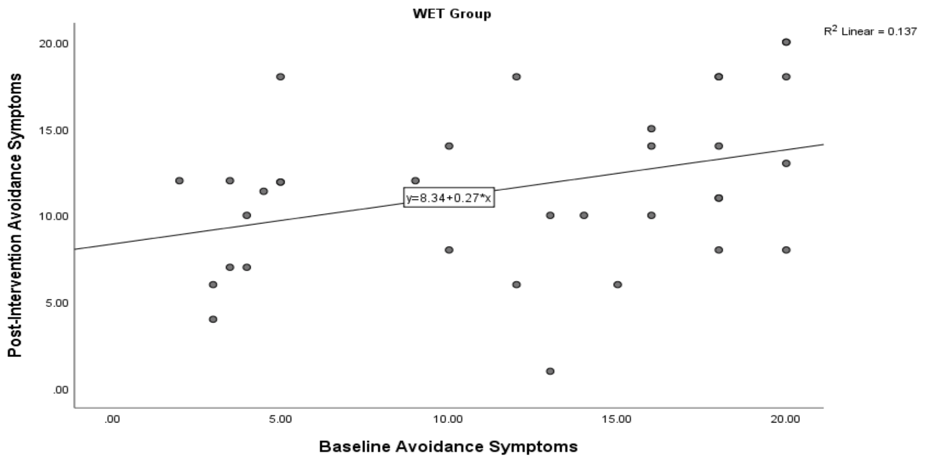

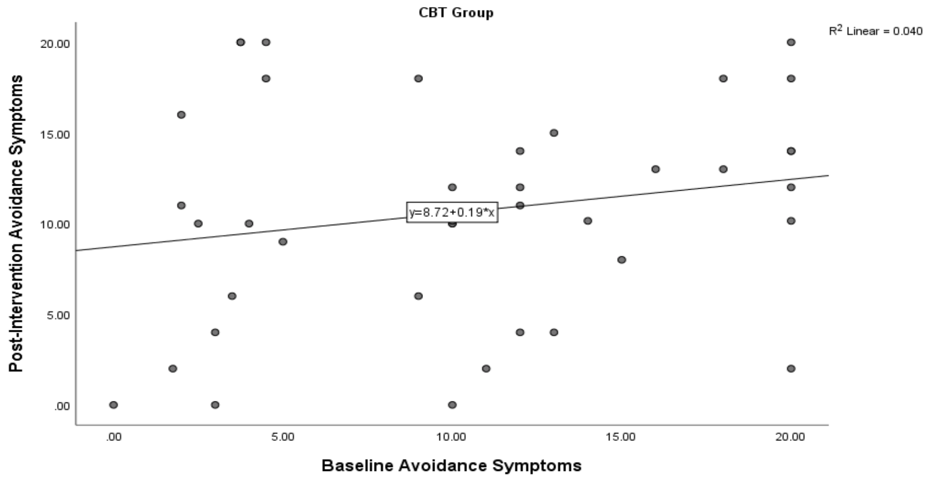

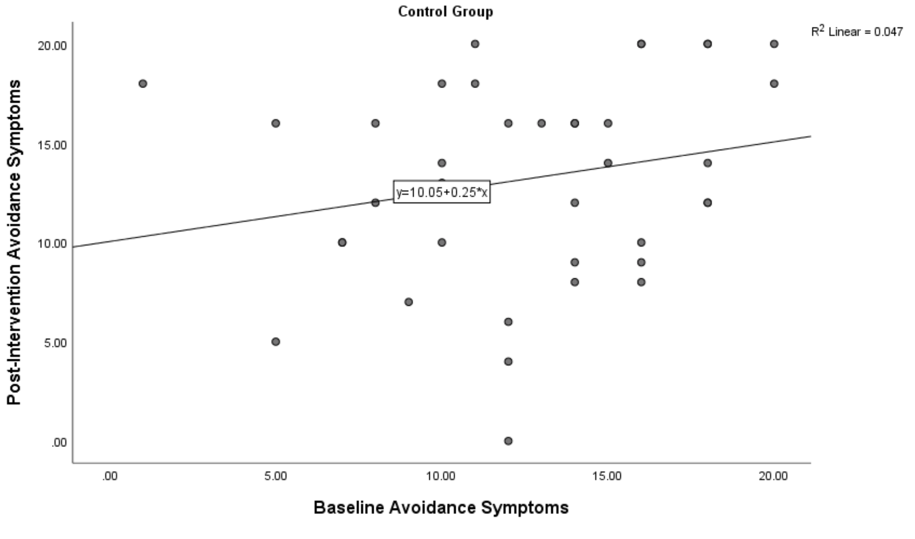

Hyperarousal Symptoms

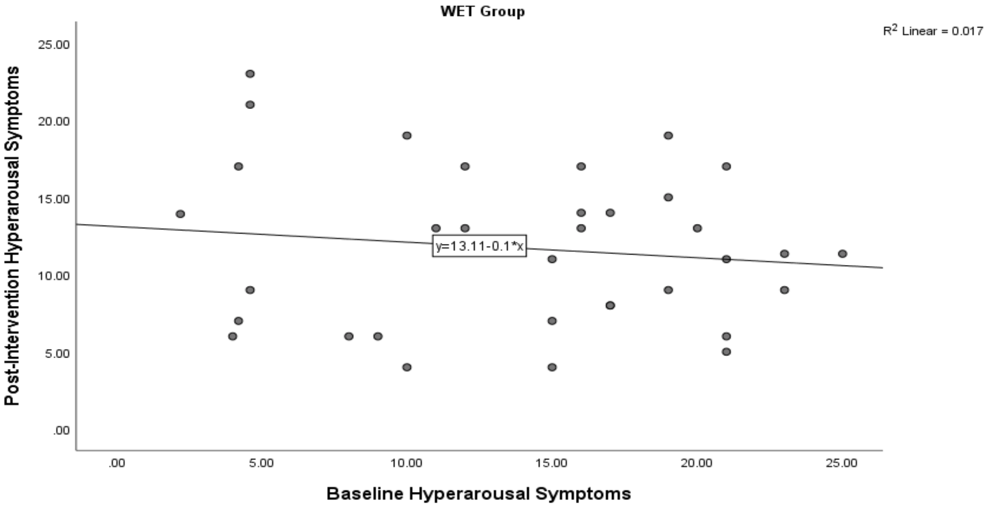

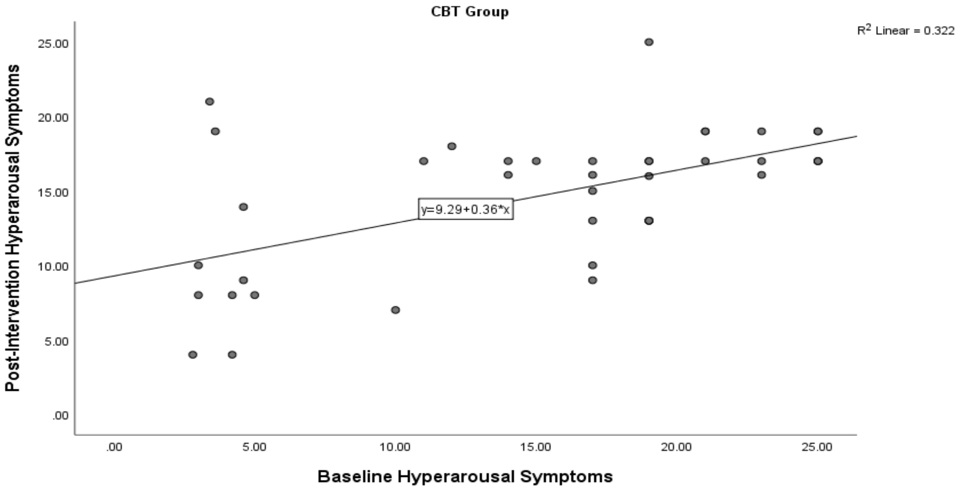

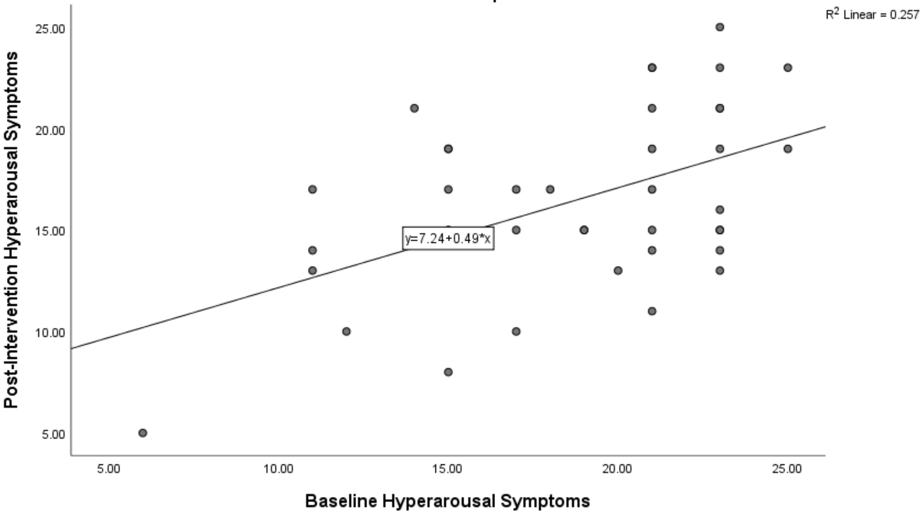

Supplement: Supplementary file 1 [file Data_Sheet_1.pdf]
